# On Medical Reform

**Published:** 1806-10

**Authors:** 


### *To the Editors of the Medical and Physical Journal.*

Gentlemen,

FROM the circular letters, which have issued through the industry of Dr. Harrison, and from the published correspondence, which appears monthly in the Medical Review; the subject of Medical Reform, and the views of the promoters of that measure, may justly be considered as at the bar of public opinion. Under which circumstances, it is presumed no offence can be given by a candid investigation of the subject itself, the object of those who favor it, and its probable interference with the privileges already exercised by the Royal Colleges of Physicians and Surgeons. Neither leisure, ability, nor inclination allow me to enter into a review of the whole of Dr. Harrison's pamphlet, nor the various answers of his different correspondents; it will be sufficient to notice his circular letter, and a few of the more prominent answers.

From the letter, we are informed, that an association of medical men, with Dr. Harrison at their head, consider the practice of Physic to have lapsed into improper hands; as the associators are competent to this discovery, it must be supposed, that they are equal to the amendment of this error, and that they consider any other hands than their own, own, unfit for the direction of the medical practice of this kingdom. With proper prudence, they consider that medical practitioners, already in possession of public confidence, will not willingly resign their claim; and therefore they provide that during the lives of the present professors, no interference is to take place with them.

How lamentable is the lot of the present generation! they are here openly told, that they are given up as fair game to a set of hungry and ignorant prescribers and dealers in medicine; but as a small consolation, they are assured their children and grand children may hope for legislative protection in the affair of health, and that their passports to futurity shall be made out only by such as are *properly licenced* to the office. Too much praise cannot be given to this philanthropic professional society for their candor and forbearance in this concession to the present professors of medicine; which can only be equalled by the succeeding paragraph of the circular letter, applying so immediately to the most failing part of human nature, namely vanity. By addressing their letters to certain persons individually, they acknowledge such persons, as regular and qualified practitioners; and farther soothe their self complacency, and gratify their private feelings, by asking whether these persons do not consider their fellow labourers in the practice of physic, and opposing candidates for medical character, as much inferior to themselves in ability; if by accident these opponents possess public esteem, whether such estimation is not very much misplaced. The answer to such questions is so obvious, that already the stage has anticipated the enquiry, in the laughable farce of the Prize, where Lenitive is introduced expatiating on the errors of Dr. Lancet, his opponent, taking to himself the quantum of praise deducted from his unfortunate antagonist. So Dr. H's correspondents are universally complainers; one is annoyed by a parson with a diploma, another by an asthma doctor, and a third by a bone-setter; all of them for some reason or other surreptitiously possest of the public confidence. As a further sacrifice to private feeling, the letter adds, that the scheme for reform being at present crude and undigested, each person addressed is not only authorised, but solicited, to send his own ideas on the method of amending the condition of physic. In times when every one neglects his own private concerns to make better the condition of his neighbour, we may judge this to be no unpleasing invitation. Accordingly, we find every correspondent ready with a plan, inlaid inlaid and dove-tailed agreeable to the rank they hold in the profession, and the interferences that particularly annoy themselves.

Then, lest the Colleges of Physicians and Surgeons should be alarmed by these revolutionary movements, we are assured that their priviledges are to be respected; a fact, which appears to be received with much hesitation by the learned body in Warwick Lane, whose late advertisement brings them and Dr. H. to issue on some part of his assertion in the circular letter; the Doctor asserting and lamenting, that the authority of the College is limited to seven miles around London, and hence the number of irregulars beyond that distance, and the crying necessity for reform; while the College asserts, on the acts of the 14th and 15th of Henry VIII. that their superintendance extends over every part of England, and implies that Dr. Harrison is himself one of those irregulars, of whom complaints have been prefered. The statement of Dr. Harrison would lead to the belief, that in London physic was administered by none but regular hands; that there the Colleges were omnipotent; that the moment a pseudo practitioner shewed his head, the Beadle of one or other College was at his heels, and the culprit was immediately summoned to the assemblage of wisdom in Warwick Lane, or Lincoln's Inn Fields, to shew cause why he presumed to assassinate His Majesty's leige subjects, proper medical licence not being first attained. How far this is true, the daily newspapers will at once decide; and the inhabitants of London will bear testimony to the liberality of those gentlemen who so amply supply him with recommendatory papers in every alley about the 'Change; the walls of which building are ornamented with the portraits of many such illustrious personages as Drs. Brodum, Sibly, &c. who equally smile at the handbill of the College of Physicians, and the circulating enquiries of Dr. Harrison.

To talk of putting the profession of physic in the country on the same footing as in London, is, for all beneficial purposes, as great a farce, as to suppose, that the Colleges exercise any of their vaunted powers over any person, not previously a member, however illiterate or ignorant, who presumes to take upon himself the medical character within the bills of mortality. Over their own members, it is true, they are specially watchful; and should any extra licentiate be found practising within the prescribed distance, there is no doubt, that he would be quickly visited by by the proper officer, to remind him of his error, and to demand the additional fees.

We, in the country, are no doubt much obliged to this liberal society of medical men, who have so kindly taken our wants into their consideration; but as the debt of gratitude is increased by the delicacy with which the favor is conferred, we cannot but be very solicitous to know of whom this society is composed; that we may judge how far their situation and abilities are equal to the accomplishment of the task they have imposed upon themselves. The letter is the avowed production of Dr. Harrison, in his character of President of the Lincolnshire Benevolent Society; and though this Society appears at the present to be forgotten by the Doctor, in the more pleasing association in Soho Square, we must look to the list of presidents of the first society to learn the names of our original benefactors. On reading over this list I cannot find, that it contains the name of any member of an English University, or of any person bearing a diploma from either the College of Physicians or Surgeons; on the contrary, several names are ornamented with the initials M. D. which were familiar to me as apothecaries, who have very lately assumed the privilege of prescribing as physicians. Consequently this society is composed of that description pointed out by Dr. Harrison, who have ventured to assume the medical profession beyond the limits of the two Colleges, because there exists no legal controul at that distance. How far chartered rights will be respected by such persons, is scarcely left to time to shew; the late revolutionary movements on the Continent having fully illustrated the mild mercies, and forbearing spirit of such self-created philanthropic societies.

Among the names of vice presidents, I observe that of Dr. Fawsset, the received partner of Dr. H, as I understand, in his medical practice; publicly avowed as such in a handbill, now before me, respecting an institution for the reception of insane persons.

I forbear to make any observation on the novelty of a medical partnership between two physicians; but I cannot be equally tolerant on the subject of the hand-bill, which was obtained by me from the White Hart Inn, Lincoln, where it adorned the walls, in the same manner that Dr. Solomon is seen to figure in the stationers' shops.

This bill states the peculiar advantages of Dr. H's institution, and the great skill of the conductors, illustrated by the case of a gentleman, who having passed under the eare care of three persons most eminent in this particular branch of the medical profession, without relief, was speedily restored to his enjoyments and his friends, by these gentlemen

If this is not a quack bill, it certainly is very much in the spirit of quackery; and it will remain for the ingenuity of Dr. Harrison to shew, how such a handbill, hung about upon the walls of inns, differs from the modest advertisements of Mr. Thomas Taylor, or Messrs. Godbolds. I have merely mentioned this fact, as the motives of men are best illustrated by their actions; and hence some light may be thrown upon the particular objects of the medical reformers.

Fearing I should intrude too much upon your valuable Journal, I conclude, though many other observations press upon my mind.

Your's, &c.

*August* 14, 1806. VERITAS.

